# Nuclear Genetic Diversity in Human Lice (*Pediculus humanus*) Reveals Continental Differences and High Inbreeding among Worldwide Populations

**DOI:** 10.1371/journal.pone.0057619

**Published:** 2013-02-27

**Authors:** Marina S. Ascunce, Melissa A. Toups, Gebreyes Kassu, Jackie Fane, Katlyn Scholl, David L. Reed

**Affiliations:** Florida Museum of Natural History, University of Florida, Gainesville, Florida, United States of America; University of Utah, United States of America

## Abstract

Understanding the evolution of parasites is important to both basic and applied evolutionary biology. Knowledge of the genetic structure of parasite populations is critical for our ability to predict how an infection can spread through a host population and for the design of effective control methods. However, very little is known about the genetic structure of most human parasites, including the human louse (*Pediculus humanus*). This species is composed of two ecotypes: the head louse (*Pediculus humanus capitis* De Geer), and the clothing (body) louse (*Pediculus humanus humanus* Linnaeus). Hundreds of millions of head louse infestations affect children every year, and this number is on the rise, in part because of increased resistance to insecticides. Clothing lice affect mostly homeless and refugee-camp populations and although they are less prevalent than head lice, the medical consequences are more severe because they vector deadly bacterial pathogens. In this study we present the first assessment of the genetic structure of human louse populations by analyzing the nuclear genetic variation at 15 newly developed microsatellite loci in 93 human lice from 11 sites in four world regions. Both ecotypes showed heterozygote deficits relative to Hardy–Weinberg equilibrium and high inbreeding values, an expected pattern given their parasitic life history. Bayesian clustering analyses assigned lice to four distinct genetic clusters that were geographically structured. The low levels of gene flow among louse populations suggested that the evolution of insecticide resistance in lice would most likely be affected by local selection pressures, underscoring the importance of tailoring control strategies to population-specific genetic makeup and evolutionary history. Our panel of microsatellite markers provides powerful data to investigate not only ecological and evolutionary processes in lice, but also those in their human hosts because of the long-term coevolutionary association between lice and humans.

## Introduction

The study of genetic diversity within and among parasite populations can provide knowledge of parasite evolutionary history, identify genes under selection, and elucidate the origin and spread of disease. Because parasites often show long-term associations with their hosts, parasites can also be used to infer host evolutionary history. In fact, several human parasites have provided insight into aspects of human evolution that were unclear from studies of direct human evidence, such as fossil or molecular data [1]–[3]. Among them, the human louse (*Pediculus humanus*) is thought to be an ancient parasite based on archeological remains, the worldwide co-distribution with humans, and coevolutionary studies [4]–[8]. This single species of louse is composed of two distinct morphological, behavioral, and ecological types: the head louse (*Pediculus humanus capitis* De Geer), and the clothing (body) louse (*Pediculus humanus humanus* Linnaeus) (see [9] for a review). Human lice are blood-sucking, wingless, host-specific ectoparasites of humans that are both obligate (cannot live off the host) and permanent (complete their life cycle on a single host species). Thus, these parasites are inextricably tied to their host in ecological and evolutionary time. Moreover, the coevolution of lice and their primate hosts over the last 25-million-year (MY) is well documented [7], [8], [10]. Although we have learned about the long-term evolutionary history of human lice, we know less about the genetic structure of living populations. The few studies that have looked at this were based on mitochondrial or a small number of nuclear DNA sequences, and those studies provided limited knowledge of the genetic structure of human louse populations worldwide. Understanding the geographic distribution of louse diversity worldwide is important for a variety of reasons, and is the focus of this study.

There are hundreds of millions of head louse infestations every year affecting mostly children of 3 to14 years of age [11]. During recent years head louse infestations have increased globally, in part because of increased resistance to insecticidal shampoos [6]. Most prominent is resistance to synthetic pyrethroids such as phenothrin and permethrin, which are the most widely used insecticides for louse control. Pyrethroid resistance in human lice is due to three knockdown (*kdr*)-type point mutations (M815I, T917I, and L920) in the voltage-sensitive sodium channel alpha-subunit gene [12], [13]. The *kdr*-type resistance has been reported in many countries among head louse populations [14], [15], and recently in clothing lice from France [16]. In order to better understand how these resistance alleles are spread, we must also have a firm understanding of neutral genetic variation and patterns of gene flow among populations of lice. In that regards, the mechanisms of how these resistance alleles spread will be improved by a better understanding of global genetic structure, which will be best revealed using highly polymorphic genetic markers like microsatellites.

Clothing lice affect predominantly homeless and refugee-camp populations [17]–[19] and are less prevalent than head lice but far more serious because they vector at least three deadly bacterial pathogens, those responsible for epidemic typhus (*Rickettsia prowazekii*), trench fever (*Bartonella quintana*), and relapsing fever (*Borrelia recurrentis*). In the last 30 years, several outbreaks of louse-borne diseases have occurred, such as epidemic typhus in Burundi, which infected over 45,000 people [20]. It once was believed that only clothing lice vectored these bacterial pathogens, however head lice have been found to carry *Bartonella quintana* in Nepal [21], the United States [22], and Ethiopia [23], [24]. Moreover, experimental infections showed that head lice can vector louse-borne diseases [25], [26]. Therefore, understanding the genetic structure of both head lice and clothing lice worldwide is of critical importance to our understanding of the risk of epidemic disease.

**Figure 1:**
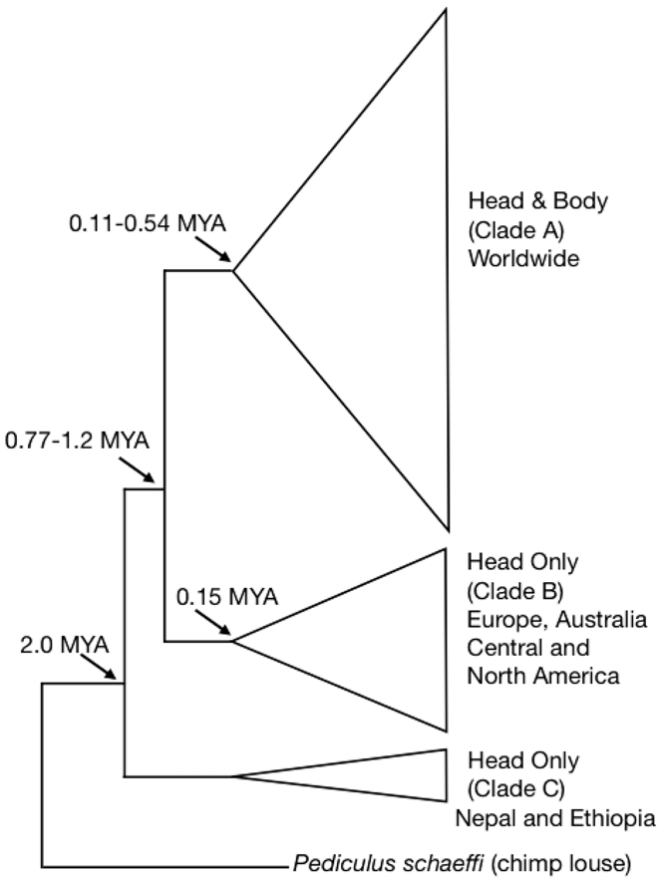
Phylogenetic relationships, timing of divergence events (in millions of years; MYA) and geographic distribution among human lice based on the mitochondrial COX1 gene [7], [27], [28]. Height of the triangles represents the number of specimens in each clade. Figure modified from [29].

The genetic diversity of human lice has been widely studied using the mitochondrial (mt) cytochrome *c* oxidase 1 gene (COX1) revealing the presence of three deeply divergent mtDNA clades or haplogroups, named A, B, and C [7], [27], [28] (Figure 1). Haplogroup A is the most common and has a worldwide distribution, whereas B and C are geographically restricted [10], [29]. Haplogroup B is found in the New World, Europe and Australia, whereas haplogroup C has only been found in Nepal and Ethiopia. Multi-Spacer-Typing (MST) markers, which included a set of four intergenic spacers, showed that lice from Clade A formed two geographic clusters, one containing all the Clade A lice outside Africa, and the other including African lice [30], [31]. Although the MST technique proved useful for differentiating African and non-African haplotype A lice, further refinement of global louse genetic structure requires markers with greater resolution. To that end we have mined the *Pediculus humanus* genome to identify new target DNA sequences to develop as microsatellite markers. We genotyped 93 human lice from 11 geographic sites distributed throughout the globe using 15 newly developed microsatellite loci. This panel of microsatellites was successful in uncovering strong signals of genetic structure that corresponded to geography and ecotype.

## Materials and Methods

### Ethics statement

The Institutional Review Board of the University of Florida exempted the study from review (Exemption of Protocol #2009-U-0422) and waived the need for written informed consent of the participants. This exemption is issued based on the United States Department of Health and Human Services (HHS) regulations. Specifically, HHS regulation 45 CFR part 46 applies to research activities involving human subjects. Because louse removal was voluntary and no information was recorded that would allow patients to be identified directly or through identifiers linked to them, the University of Florida waived the need for written informed consent from the participants.

### Sampling

Sampling details are provided in Methods S1. Briefly, 75 human head lice were collected from different individuals at 10 localities throughout the world (Table 1). Clothing lice came from two sites: Canada and Nepal. Canadian clothing lice (N = 16) were collected from a single homeless person. The two clothing lice from Nepal (N = 2) were collected from two persons.

**Table 1:** Human louse samples used in the current study.

World region	Country	Site Code	Head lice		Clothing lice		Facility		City, State or Province			Collectors		
North	Canada	CAN		16		Homeless shelter		Lethbridge, Alberta			D. D. Colwell			
America	USA	Oce	25			Louse comb facility		New York Area, New York			C. Gilbert			
		SF	1			Louse comb facility		San Francisco, California			M. Mitchell			
		Martin	1			Louse comb facility		Martin County, Florida			K. Shepherd			
		WPB	1			Louse comb facility		West Palm Beach, Florida			K. Shepherd			
							
Central America	Honduras	Hon	20			Orphanage		San Francisco, Zamorano Valley			K. Shepherd			
							
Asia	Thailand	Th	1			Orphanage		Sanklaburi, Kanchanaburi			K. Shepherd			
	Nepal	Nepal	1	2		Unknown		Kathmandu			K. Yoshizawa			
	Cambodia	Cam	22			Orphanage		Batambang, Batambang			K. Shepherd			
							
Europe	Norway	Nw	1			Unknown		Tromso			K. Gravningen			
	United	UK	1			Unknown		Surrey, England			N. Hill			
	Kingdom		1			Unknown		Dumfries & Galloway, Scotland			N. Hill			
Total			75	18										

### Data harvesting, screen for repeat motifs and primer design

**Figure 2:**
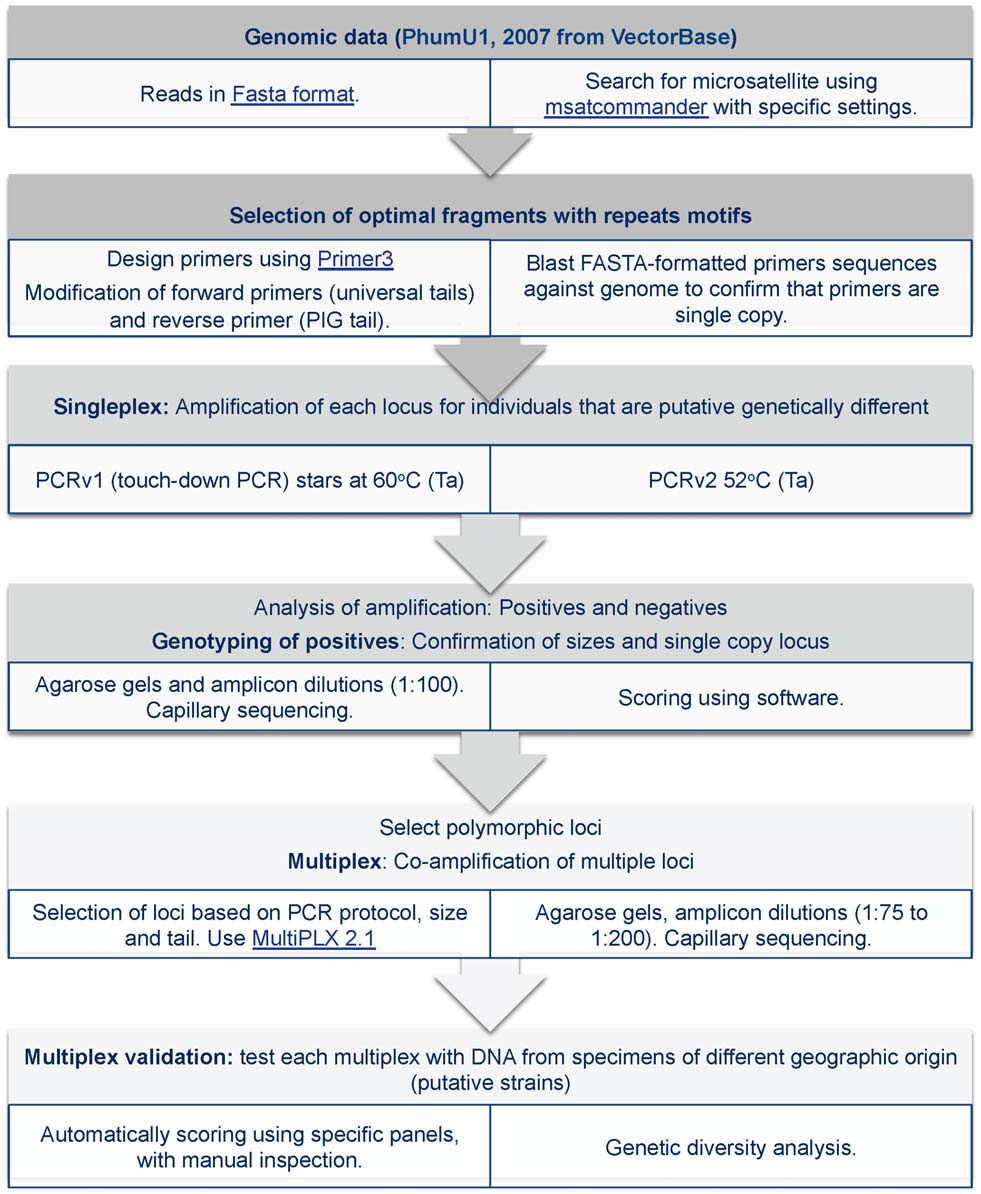
Analytical pipeline used to develop microsatellite loci from genomic data including the development of multiplexes using multiple fluorescently labeled universal primers. Ta: Annealing temperature. (see Methods S1 for details and references).

An overview of our primer development strategy and multiplex optimization is presented in Figure 2 and we included a detailed description for microsatellite development in Methods S1. Briefly, assembled genome sequence data for *Pediculus humanus* USDA strain (PhumU1, 2007) were obtained from VectorBase (http://www.vectorbase.org/) [32], [33] and screened for tandem repeat motifs. Candidate sequences were subject to primer design using Primer3Plus [34].

### Multiple co-amplification: Multiplexes

Primer pairs that produced amplicons of the expected size and demonstrated allelic variation were selected for further testing and multiplex optimization (Table S1). We used fluorescently labeled universal primers: M13 (5’-CACGACGTTGTAAAACGAC-3’) and CAG (5’-CAGTCGGGCGTCATCA-3’), each labeled with a unique fluorescent tag (e.g. FAM, VIC, NED, PET) to co-amplify multiple loci. Locus-specific primers were modified by adding the matching 5′ universal primer sequence tails.

### Microsatellite diversity analysis

The final optimized four multiplexes were tested on the DNA extracted from 93 human lice from 11 sites (Table 1; Methods S1). Evidence for large allelic drop out and null alleles was determined using Microchecker version 2.2.3 [35]. We used the software Arlequin version 3.5.1.2 [36] to determine number of alleles per locus, observed and expected heterozygosity (H_O_ and H_E_), and mean *F*_IS_ estimates (an index of the inbreeding of individuals resulting from the non-random union of gametes within a subpopulation) over loci per populations. Confidence intervals for *F*_IS_ estimates were calculated by bootstrapping over loci using 1,023 random permutations. Genotypic disequilibrium among loci was estimated using FSTAT version 2.9.3.2 [37], [38].

### STRUCTURE analysis

Population structure was inferred with a Bayesian clustering approach implemented in the STRUCTURE software [39] (http://pritch.bsd.uchicago.edu/structure.html). This method uses the individual multi-locus genotypic data to evaluate models assuming different numbers of genetic clusters (*K*) based on the posterior probabilities given the data and model. All simulations used 50,000 Markov chain Monte Carlo (MCMC) generations in the burn-in phase and 100,000 generations in the data collection phase. Ten independent runs, using default parameters for each *K*, to ensure equilibration during burn-in and consistency in estimation of the posterior probabilities. Selection of the number of distinct clusters was based on the evaluation of the Δ*K* statistic [40]. The ten STRUCTURE runs at each *K* produced nearly identical individual membership coefficients. The run with the highest likelihood of the data given the parameter values for the predominant clustering pattern (i.e. the mode) at each *K* was used for plotting with DISTRUCT [41] (http://www.stanford.edu/group/rosenberglab/distruct.html). A series of STRUCTURE analyses were conducted: S1) the worldwide dataset for all 15 loci, S2) the worldwide dataset for 14 loci (we excluded locus M2-13 due to some missing data), S3) the worldwide dataset for 14 loci plus the mitochodrial haplogroup coded as another marker, and S4) local datasets for sites with 10 or more lice (Canada, New York, Honduras and Cambodia). We ran up to *K* = 15 for the global (S1, S2 and S3), and *K* = 5 for the local (S4) datasets. The mitochondrial haplogroup information was obtained from Ascunce et al. (in prep.).

### Pairwise estimates of population structure and gene flow

Principal coordinate analysis (PCA) was conducted using pairwise genetic difference between individuals calculated in GenAlEx [42], [43] (http://biology.anu.edu.au/GenAlEx) to validate and further define genetic clusters for these lice. For sample sizes exceeding 10 lice, we also estimated population pairwise values of the Weir and Cockerham (1984) [44] analogue of *F*_ST_, Θ_WC_, in FSTAT 2.9.3.2 [35], [36] and gene flow (*N_e_m*) based on the private alleles method [45], as implemented in the online program Genepop version 4.0.10 [46], [47] (http://genepop.curtin.edu.au/). These two estimates: Θ_WC_ and *N_e_m* were also evaluated considering the genetic clusters inferred from STRUCTURE simulations.

### Selection test

To detect outlier loci under selection we used the program BayeScan [48] (http://cmpg.unibe.ch/software/bayescan/). BayeScan is a hierarchical Bayesian method that assumes that allele frequencies within populations follow a multinomial-Dirichlet distribution [49]–[51]. It estimates population-specific *F*_ST_ coefficients, therefore allowing for different demographic histories and different amounts of genetic drift between populations. BayeScan incorporates the uncertainty on allele frequencies due to small sample sizes. The estimation of model parameters was automatically tuned on the basis of 20 short pilot runs of 5,000 iterations. The sample size was set to 5,000 and the thinning interval to 10, resulting in a total chain length of 100,000 iterations. Four independent runs were performed for each of the two datasets to account for the consistency of the detected outliers. The loci were ranked according to their estimated posterior probability and all loci with a value over 0.76 were retained as outliers. This corresponds to a Bayes Factor >3, which provides substantial support for acceptance of the model [52].

### Relatedness

The average relatedness (*r*) among lice was determined using the software Relatedness version 5.0 [53] (http://www.gsoftnet.us/GSoft.html) for the following groups of lice: R1) clothing lice from a single homeless person in Canada; R2) head lice from people in New York; and a single head louse from each child in one of two orphanages in: R3) Cambodia, and R4) Honduras.

## Results

### Microsatellite Diversity

We found a total of 295,733 di-, tri- and tetranucleotide tandem repeat motifs sequences. Tri-nucleotide motifs are the most abundant microsatellites, making up 62% of perfect microsatellites (Figure S1). Comparative genomic studies have revealed a great heterogeneity in microsatellite abundance and composition across taxa. Particularly, some other arthropods such as *Aedes aegypti* [54] have also shown skews toward tri-nucleotide repeats.

**Figure 3:**
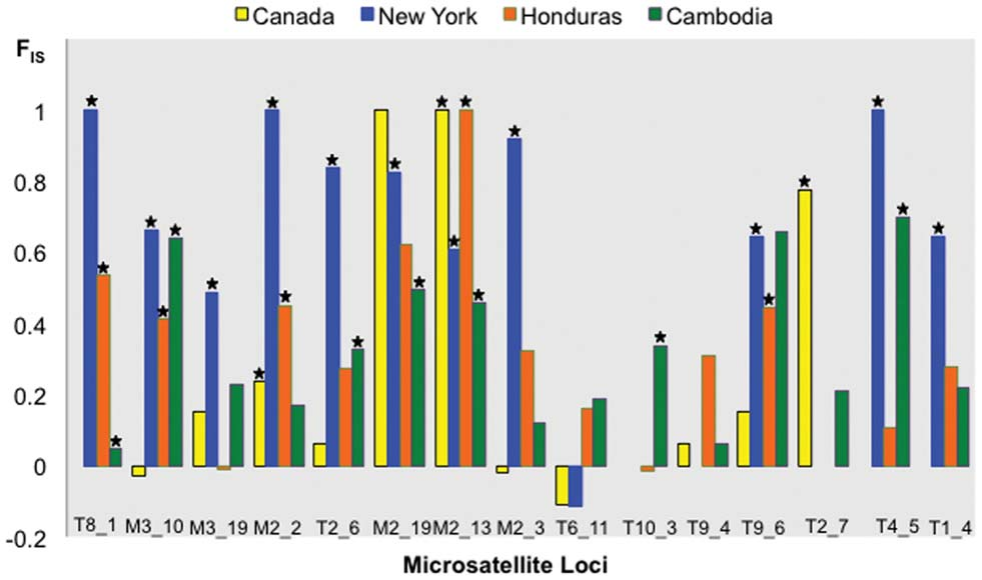
*F*_IS_ estimates by locus within populations. For monomorphic loci estimates were not calculated and they are shown in the graphs as gaps. Stars indicate significant departure from Hardy-Weinberg equilibrium (*P*<0.05).

From the approximately 150 primer pairs tested, 33% gave clear amplifications and showed allelic diversity. A final set of 15 microsatellite loci were thoroughly optimized and validated over 93 human lice from around the world (Tables S1, S2, and S3). All microsatellite loci were highly polymorphic, with an average of 10 alleles per locus and an average H_O_ and H_E_ of 0.2748 and 0.7136, respectively (Table 2). In populations with more than 10 lice (Canada, New York, Honduras and Cambodia), Micro-Checker analysis found no evidence for scoring errors due to stuttering or large-allele dropout for each locus in each population. However, Micro-Checker identified null alleles in multiple loci at each geographic location. In these same populations, genotype proportions deviated significantly from Hardy-Weinberg expectations due to heterozygote deficiencies (Table S4). Values of *F*_IS_ were significantly positive for a large number of loci, indicating also heterozygote deficiencies within these populations (Figure 3). Overall all four sites showed high values of *F*_IS_ from 0.232 in Canada to 0.767 in New York (Table 2). Moreover, H_O_ and *F*_IS_ were significantly correlated within New York and Cambodia populations (Spearman rank correlation tests; *P*<0.001). Out of 519 tests for linkage disequilibrium, none of the tests were found to be significant after Bonferroni correction (adjusted at the 5% level; *P*<0.000043). We excluded M2-13 loci from further analyses because of its relatively high percentage of missing data (26%).

**Table 2:** Microsatellite polymorphisms among human louse populations.

Continent/Subcontinent	Country	Site Code	N	AA	H_O_	H_E_	*F*_IS_
North America	Canada	CAN	16	2.889	0.347	0.448	0.165
							
	USA	Oce	25	2.900	0.083	0.352	0.787*
		SF	1	1.000	n/a	n/a	n/a
		WPB	1	0.929	n/a	n/a	n/a
		Martin	1	1.500	n/a	n/a	n/a
							
Central America	Honduras	Hon	20	4.417	0.365	0.567	0.319*
							
Asia	Thailand	Th	1	1.357	n/a	n/a	n/a
							
	Nepal	Nepal	3	3.167	0.555	0.722	0.273
							
	Cambodia	Cam	22	6.846	0.483	0.703	0.289*
							
Europe	Norway	Nw	1	1.071	n/a	n/a	n/a
	United Kingdom	UK	2	1.286	0.167	0.611	0.800
Total/Average			93	10.200	0.275	0.714	0.374*

*Note*: N refers to total sample size, AA is the average number of alleles, H_O_: mean observed heterozygosity; H_E_: mean expected heterozygosity; *F*_IS_: inbreeding coefficient. n/a indicates values not estimated due to sample size.

* denotes significant deviation from Hardy-Weinberg expectations at *p*<0.05; positive value indicates heterozygote deficit.

### Nuclear genetic clusters at the worldwide scale

**Figure 4:**
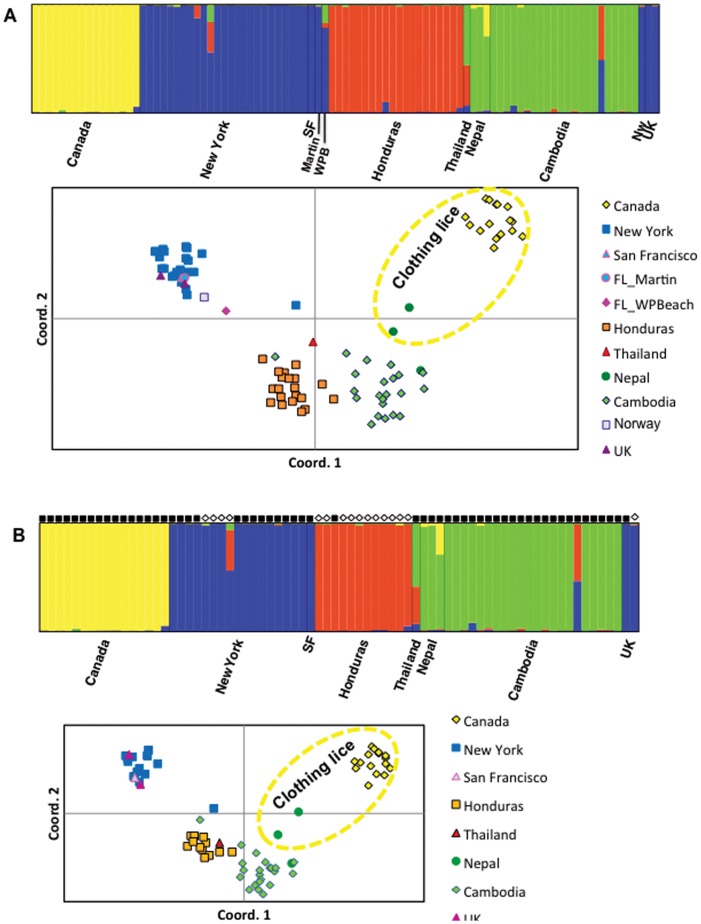
Genetic clusters inferred from STRUCTURE simulations (*K* = 4) for each dataset. A) the worldwide dataset for 14 loci (we excluded M2-13 due to missing data), and B) the worldwide dataset for all 14 loci plus the mitochondrial haplogroup coded as an additional locus. In the bar plot, each individual is represented by a single vertical line and the length of each color segment represents the proportion of membership (*Q*) to the four clusters. In pannel B, for each louse sample we added the mitochondrial haplogroup as filled black squares for Clade A and the open diamonds representing Clade B. Distributions of points in the first two dimensions resulting from principal coordination analyses (PCA) conducted using pairwise genetic distance comparisons of the same dataset used for the STRUCTURE analyses are below each STRUCTURE plot.

Population structure was inferred with a Bayesian clustering approach implemented in the STRUCTURE software [39]. In all the three STRUCTURE analysis, all worldwide human lice were assigned to four genetic clusters (*K* = 4), one defined by clothing lice from Canada, the other head lice from North America and Europe, a third cluster was composed of head lice from Honduras, and the fourth cluster included Asian lice (both head and clothing lice) (Figure 4A) (STRUCTURE with 15 microsatellite loci is not shown). In another STRUCTURE analysis, we incorporated the mitochondrial haplogroup data for each louse (Figure 4B). This STRUCTURE analysis showed that there is no correlation between mitochondrial haplogroups (A and B) and nuclear genetic clusters, at least among the current samples. We also employed the multivariate technique Principal Coordinate Analysis (PCA) implemented in GenAlEx [42], [43] that allows the visualization of the spatial distribution of the genetic differences among all samples. We found similar results as the STRUCTURE analyses, where one cluster included head lice from North America and Europe, while all Asian and Central American lice comprised a second cluster with the exception of the clothing louse from Nepal that showed an intermediate position between this group and clothing lice from Canada (Figure 4).

### Nuclear genetic clusters at the local/cluster scale

**Figure 5:**
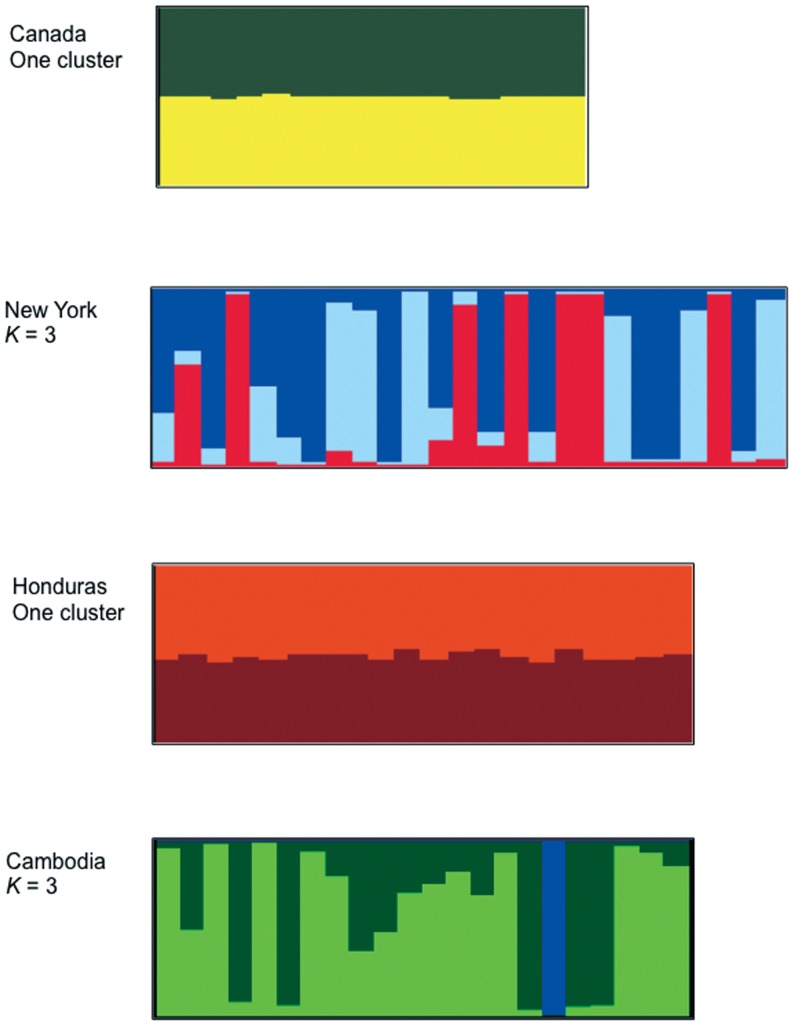
STRUCTURE results for each geographic site with 10 or more lice: Canada, New York, Honduras and Cambodia (based on average membership coefficient, *Q*, derived from 14 microsatellite loci).

For Canada, New York, Honduras, and Cambodia populations we further analyzed their genetic substructure by analyzing each population individually using STRUCTURE. These results revealed an increase in the number of regional genetic clusters in New York (*K* = 3), and in Cambodia (*K* = 3) (Figure 5). Although Evanno's method cannot evaluate *K* = 1 as the most likely number of clusters, we found that populations from Canada and Honduras showed admixture for all individuals when *K* = 2. This can be interpreted as evidence supporting Canada and Honduras as a single genetic cluster, respectively, at least with the current number of microsatellite markers analyzed.

### Pairwise estimates of population structure and gene flow

In order to estimate gene flow, we calculated *F*_ST_ and *N_e_m* across human louse populations with more than 10 lice (Table S5). Both *F*_ST_ and *N_e_m* produced results concordant with the STRUCTURE analysis. There were high values of genetic differentiation (*F*_ST_, above 0.4087) between the clothing louse population (Canada) and any of the remaining head louse populations. However, these results should be taken with caution and more clothing louse populations should be analyzed to corroborate these results. Effective rates of nuclear gene flow (*N_e_m*) using the private allele method yielded values less than 1.0. The highest value was found between Central America and Asia (0.7961), which may represent a more recent ancestry.

### Selection test

The microsatellite data set was used in a global analysis for outlier detection using BayeScan [47]. A single locus (T2_7) showed evidence of balancing selection with a posterior probability of 0.9962, log_10_ (PO) of 2.4185. This corresponded to a Bayes Factor larger than 100, which provides ‘decisive’ evidence for selection [52].

### Relatedness

For the fine scale genetic structure analyses we examined the patterns of relatedness (*r*) among clothing lice from a single homeless person, and head lice from each population. These analyses can determine whether a few genotypes are responsible for the production of the majority of offspring. Although average population relatedness values (*r*) were negative suggesting outbreeding and unrelated populations [53] (Table 3), a detailed analysis of pairwise comparison between individuals within each site showed a variable range of relatedness (Figure S2). Roughly, 50% of lice in each site had *r* values lower than 0, thus they are very different from each other. The second largest group of lice had *r* values up to 0.4, showing some relatedness. All sites showed the presence of some full-siblings (*r* = 0.5) and the highest level of inbreeding with identical genotypic profiles was found in New York (*r* = 1). These high relatedness values found in the New York samples parallel the evidence of heterozygote deficits and high inbreeding, reflecting a biological phenomenon.

**Table 3:** Whole population relatedness values (*r*).

Site	*r*	*p*
R1: clothing lice from single shelter (Canada)	−0.0667	0.0000*
R2: head lice from New York area (Oce)	−0.0417	0.0000*
R3: head lice from single orphanage (Cambodia)	−0.0486	0.0017*
R4: head lice from single orphanage (Honduras)	−0.0534	0.0011*

Note: *indicate statistical significance at *p*<0.05.

## Discussion

### Life history traits and genetic diversity

Understanding the processes that shape the genetic structure of parasite populations is critical in predicting how an infection can spread through a host population and for the design of effective control methods. Here, we provide the first assessment of genetic structure in human louse populations from around the world using microsatellite loci. It is known that parasite life history strategies, such as dispersal and mode of transmission, directly affect their genetic structure. In our study, a large number of loci showed significant heterozygote deficits relative to Hardy–Weinberg equilibrium plus high *F*_IS_-values (Table 2, Table S4). This genome-wide pattern would suggest that inbreeding is common in human lice, a pattern expected given its parasitic life history. In an earlier study using five microsatellite loci analyzing head and clothing lice from doubly-infested individuals [55], the authors also found that certain loci were consistently out of Hardy-Weinberg equilibrium reflecting a population-specific phenomenon rather than microsatellite locus-specific issue. Alternatively, deficiency of heterozygotes could also been explained by null alleles, however we successfully mapped all PCR primers used in this study to assembled genomes from clothing lice, mtDNA Clade A head lice, and mtDNA Clade B head lice, and found no nucleotide mismatches at our priming sites from the clothing louse genome and only one mismatch in each of three primers from the head louse genomes. The Wahlund effect (population substructure) could also cause significant heterozygote deficits relative to Hardy–Weinberg equilibrium. Indeed, in New York three genetic clusters were detected through STRUCTURE analysis.

Interestingly, we found a weaker genome-wide effect in terms of heterozygote deficits relative to Hardy–Weinberg equilibrium and null alleles in clothing lice than among head lice (Table 2, Table S4). Some life history traits of clothing lice may lead to higher effective population sizes (*N_e_*) than in head louse populations. For example, clothing louse females lay up to 300 eggs along the seams or hems of clothes compared with 150 eggs laid (and glued) on human hair shafts by head louse females.

Another factor that can contribute to the difference in heterozygosity is selection for insecticide resistance. Among head louse populations, intense selection by insecticides may have resulted in periodic population bottlenecks. These demographic events are expected to have genome-wide effects by reducing genetic polymorphisms in the louse genome. Whereas insecticide resistance is well known for head lice, it is less common in clothing lice and only recently has a study performed *kdr*-allele typing for clothing lice from France [16]. Further studies including sympatric clothing and head louse populations are needed to discern the role of these factors in shaping the genetic structure and diversity of human lice.

### Gene flow and the evolution of insecticide resistance

Gene flow can have broad implications in the evolution of drug resistance. For example, the widespread resistance to the most common antimalarial drugs (chloroquine and sulfadoxine-pyrimethamine) is thought to be the result of few drug-resistance alleles coupled with high gene flow in vectors (mosquitoes) and human hosts [56]–[58]. Alternatively, convergent insecticide resistance phenotypes can evolve through identical or different mutations in independent populations. For example, the same mutations that confer insecticide resistance in the E3 esterases of blowflies and houseflies can arise *de novo* and be selected for in separate populations [59]. In human lice, because of the strong genetic structure with low gene flow among populations, it seems more likely that human lice will follow a model of parallel adaptive evolution, where new resistance alleles evolve in different populations. However, the study of the evolution of resistance in human lice is in its infancy and many basic questions still need to be addressed. Pyrethroid resistance occurs worldwide with variable resistant *kdr*-like haplotype frequencies in each geographic area [14], [15]. Moreover, a recent study highlighted that some *kdr*-alleles were not correlated with treatment failure, thus other factors may be involved [60]. In other insects, functional genomic analysis has shown that insecticide resistance could constitute a multigenic trait involving large parts of the insects' genome [61]–[63]. To test the model of resistance evolution in human lice, regional comprehensive studies are needed combining phenotypic and genotypic analysis of resistance coupled with neutral markers, including microsatellite loci linked to the voltage-sensitive sodium channel alpha-subunit gene as well as other genes involved in resistance. Interestingly, with our microsatellite markers panel, we detected one outlier locus that could be mapped at a unique location in the reference genome and was in the vicinity of a putative gene coding for a carbonic anhydrase enzyme. These enzymes seem to be involved in pH regulation (alkalization mechanisms) of a lepidopteran caterpillar and larval mosquito guts [64]–[66]. Thus, we suggest that this gene is a promising candidate for future functional analysis.

### Clothing and head louse differentiation

The species status of *P. humanus* (whether head and clothing lice represent one species or two) has been a topic for debate for over a century (see [9] for a review). However, the difficulty in determining whether a group of organisms constitutes an independently evolving lineage is particularly compounded in parasites. In our study, the clothing lice (Canada and Nepal) grouped more closely with the Central America-Asia cluster probably because of close ancestry (Figure 4). These results are consistent with the idea that clothing lice evolved from head louse ancestors, invading the body region only recently with the advent of clothing use in modern humans [7], [27], [28]. Further, studies have shown that clothing lice emerged from only one of the three mitochondrial haplogroups (Clade A) roughly 83,000 years ago [67]. Although clothing lice belong to a single mtDNA clade, they appear to have evolved locally (*in situ*) throughout the world from head louse populations [31]. In contrast, Leo et al. (2005) used five microsatellite loci to address the issue of species status within *P. humanus* by analyzing head and clothing lice from doubly-infested individuals [55]. The authors found that head and clothing lice from each human host formed separate nuclear clusters, and each host represented a unique genetic cluster [55]. Their conclusion should be taken with caution due to the small sample size, restricted geographic distribution of the samples and few loci used in their study. In our study, we are also puzzled by the distant genetic relationship between Canada (clothing) and New York (head) lice. Could the strong pyrethroid-based pediculicide treatment of head lice in developed countries [15] account for the large differentiation found in this study between clothing lice from Canada and head lice from New York? Could that be the case for the louse population from China and Nepal studied by Leo et al. (2005) [55]? Understanding the presence or absence of gene flow between head and clothing lice is of paramount importance, because in addition to clothing lice, researchers are now finding an increasing number of cases of louse-borne bacteria in head lice.

### Human louse genetic diversity and human evolution

**Figure 6:**
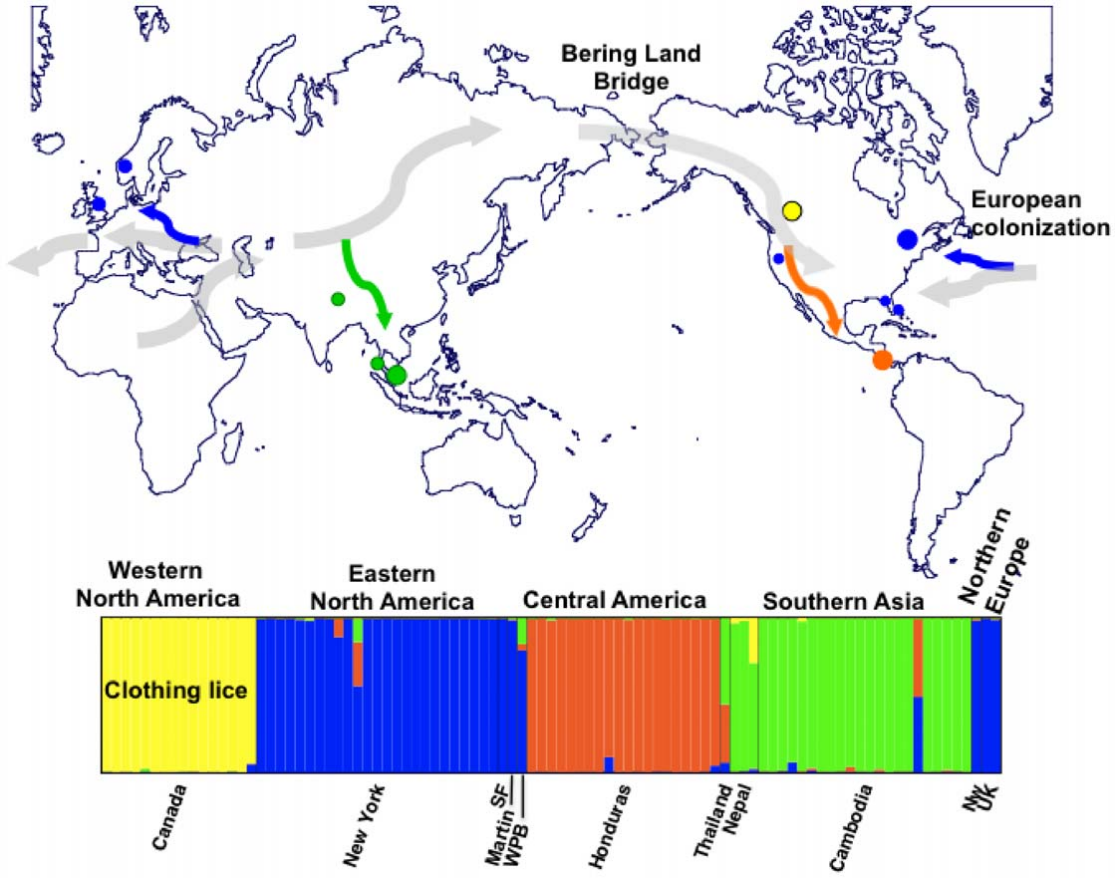
Map depicting the geographic distribution of the nuclear genetic diversity among the human louse populations included in this study. Colored circles on map indicate collecting sites, with the color of each circle corresponding to the majority nuclear genetic cluster to which sampled individuals were assigned. Large colored circles are sites with 16 or more lice, small colored circles represent sites with one to three lice. Thick grey arrows indicate proposed migrations of anatomically modern humans out of Africa into Europe, Asia and the Americas, as well as the more recent European colonization of the New World. Colored arrows represent hypothetical human louse co-migrations. The bottom panel is the plot from STRUCTURE corresponding to the assignment of 93 lice from 11 geographical sites (from Figure 4A).

One of the more exciting aspects of our understanding of nuclear diversity in lice is its application to human evolution. Bayesian clustering analyses assigned lice to four distinct genetic clusters: the first cluster consisting of clothing lice from Canada, the other cluster included head lice from North America and Europe, a third cluster was composed of head lice from Honduras, while the fourth cluster included Asian lice. How this geographic structure reflects human migrations requires greater sampling. Although preliminary, our study suggests that the Central America-Asian cluster is mirroring the (human host) colonization of the New World if Central American lice were of Native American origin and Asia was the source population for the first people of the Americas as has been suggested (Figure 6) [68], [69]. The USA head louse population might be of European decent, explaining its clustering with lice from Europe. Within the New World, the major difference between USA and Honduras may reflect the history of the two major human settlements of the New World: the first peopling of America and the European colonization after Columbus (Figure 6).

This study provides preliminary evidence that microsatellites are effective in determining population genetic structure among human louse populations at a scale that could help us better understand patterns of human migration worldwide and might also provide insight into interactions between archaic hominids and anatomically modern humans, *Homo sapiens*. For example, previous studies [7] suggested that louse mtDNA haplogroup A has had a long history associated with the host lineage that led to anatomically modern humans, *Homo sapiens* (Figure 1). Studies of modern human expansion out of Africa show the footprint of serial founder effects on the genetic diversity of human populations as revealed by the human pattern of increased genetic distance and decreased diversity with distance from Africa [69]. The microsatellite loci developed in this study are ideal markers to measure louse genetic diversity and how it parallels to human diversity. Louse mitochondrial haplogroup B is found in the New World, Europe and Australia but not in Africa. Reed et al. [7] suggested that its evolutionary origins might lie with archaic hominids from Eurasia (i.e., *Homo neanderthalensis*) and that they became associated with modern humans via a host switch during periods of overlap. If true, then examining nuclear markers for lice with haplotypes A (host: *H. sapiens*) and B (past host: *H. neanderthalensis*; current host: *H. sapiens*) would permit a test for admixture in lice and could provide a time frame for when louse admixture occurred (i.e., host switching). This host switching would give us a time period during which *H. sapiens* and *H. neanderthalensis* co-occurred. Whether modern humans interbred with archaic hominids is a much-debated question [70]–[75]. However, genomic evidence using ancient DNA from Neanderthals and modern DNA from living humans suggests that interbreeding might have occurred between 47,000 and 65,000 years ago [76]. A less clear question is whether anatomically modern humans overlapped or interbred with other archaic hominids in Asia or Africa. Recent genetic studies have also supported both Asian and African archaic admixtures. If the older haplogroup C lice evolved on archaic hominins in Asia or Africa, then the study of haplogroup C lice could also provide compelling evidence of close-proximity interactions of modern and archaic hominins in Asia or Africa.

## Conclusion

Understanding the processes that shape the genetic structure of human parasite populations is important to both basic and applied evolutionary biology. In human lice, knowing the processes and mechanisms that maintain and generate human louse genetic diversity within and among host populations is critical for our ability to design effective control methods and to predict how louse-borne diseases can spread through human populations. In this study, we showed that human louse populations are genetically structured based on geography (Figure 6). The high degree of genetic structure may lead to the evolution of different resistance alleles among different populations, thus suggesting the need of regional epidemiological studies to control lice. Furthermore, this work has shown that the study of the genetic diversity of human lice could help us better understand patterns of human migrations worldwide at different temporal scales, and could also be used to test hypotheses about human evolution such as ecological interactions between modern and archaic hominins.

## Supporting Information

Figure S1 **Microsatellite abundance (counts): Data are shown for di- (diagonal lines), tri- (grey), and tetra- (solid black) per number of repeat motifs.**
(PDF)

Figure S2 **Average pairwise relatedness (** ***r*** **) of lice within sites.** Relatedness values can range from 1 (individuals are identical for all alleles assessed) to –1 (individuals have no alleles in common).
(PDF)

Methods S1 **This document includes: Supporting Information Methods and Supporting Information References.**
(XLS)

Table S1 **Characteristics of 27 microsatellite loci developed using the genome data of clothing louse (PhumU1, 2007).**
(XLS)

Table S2 **Master mixes of primers for each multiplex. All primers working solutions are 10 µM.**
(XLS)

Table S3 **Total PCR mix, quantities are indicated for individual tubes and for whole plates.** Labeled tail primer concentration is 10 µM.
(XLS)

Table S4 **Polymorphisms of the microsatellite loci used in this study.**
(XLS)
