# Supplementary figures and images for: Nuclear Genetic Diversity in Human Lice (Pediculus humanus) Reveals Continental Differences and High Inbreeding among Worldwide Populations
Source: PLoS One. 2013 Feb 27;8(2):e57619. doi: 10.1371/journal.pone.0057619 (PMC3583987; doi:10.1371/journal.pone.0057619)

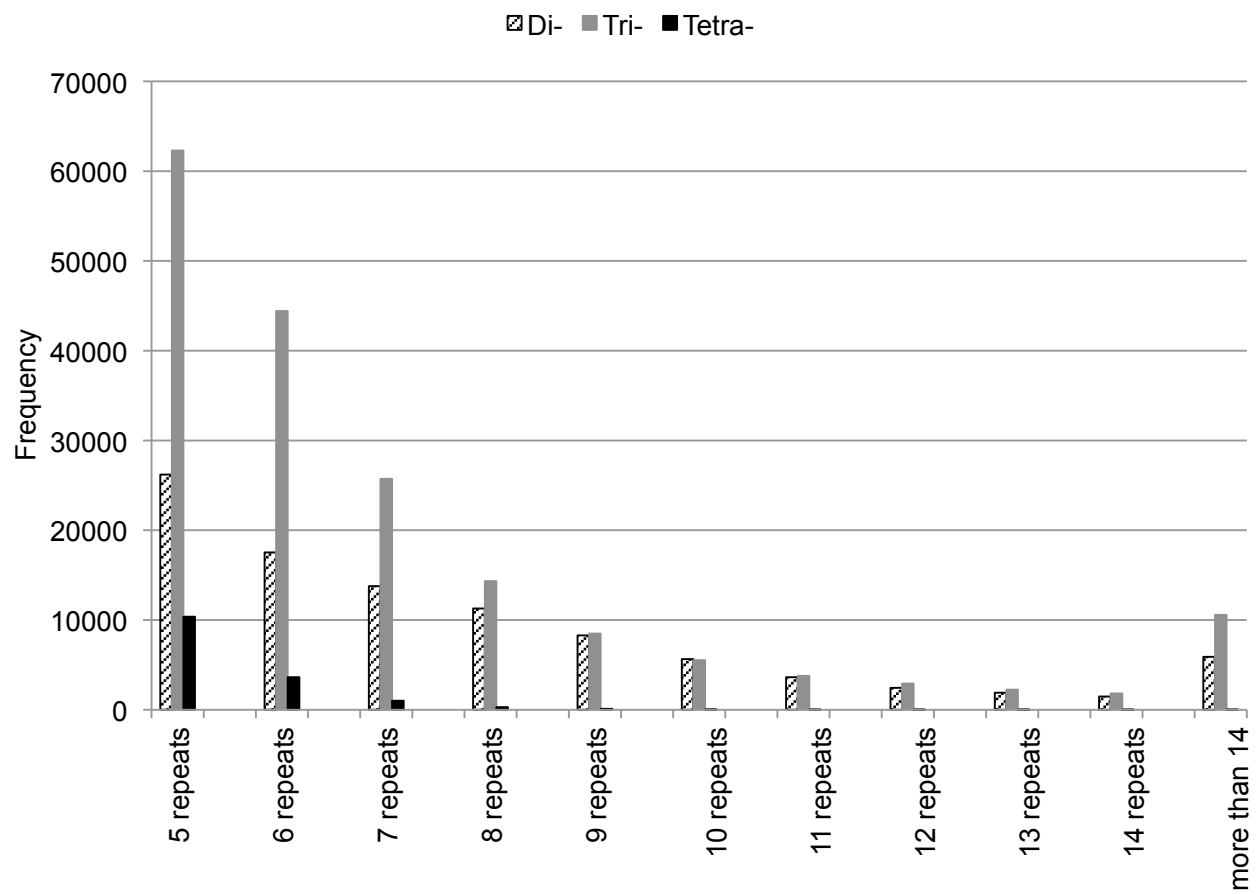

Supplement: Figure S1 — Microsatellite abundance (counts): Data are shown for di- (diagonal lines), tri- (grey), and tetra- (solid black) per number of repeat motifs. (PDF) [file pone.0057619.s001.pdf]

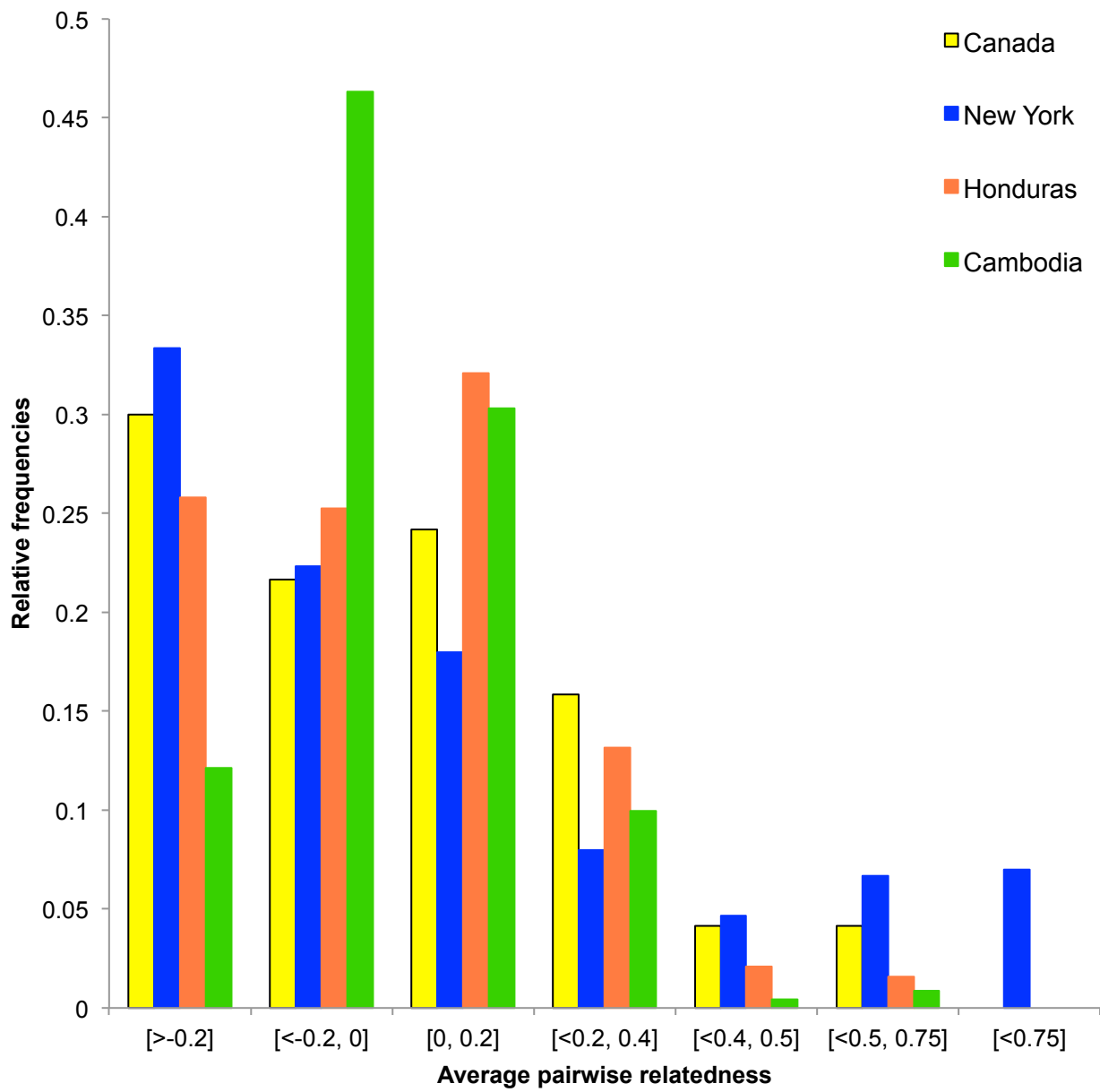

Supplement: Figure S2 — Average pairwise relatedness ( r ) of lice within sites. Relatedness values can range from 1 (individuals are identical for all alleles assessed) to –1 (individuals have no alleles in common). (PDF) [file pone.0057619.s002.pdf]
