# Supplementary material for: Nuclear Genetic Diversity in Human Lice (Pediculus humanus) Reveals Continental Differences and High Inbreeding among Worldwide Populations
Source: PLoS One. 2013 Feb 27;8(2):e57619. doi: 10.1371/journal.pone.0057619 (PMC3583987; doi:10.1371/journal.pone.0057619)
Supplement: Methods S1 — This document includes: Supporting Information Methods and Supporting Information References. (XLS) [file pone.0057619.s003.xls]

***PLOS ONE***

**Supporting Information Section**

**Nuclear genetic diversity in human lice (*Pediculus humanus*) reveals continental differences and high inbreeding among worldwide populations**

Marina S. Ascunce, Melissa A. Toups, Gebreyes Kassu, Jackie Fane, Katlyn Scholl, **and David L. Reed**

**This PDF includes:**

**Supporting Information Methods**

**Supporting Information References**

**Supporting Information Methods**

***Laboratory Procedures***

***Sampling***

Head lice were collected mostly from school-age children. Each child’s head was visually inspected, and head lice were removed using a fine-toothed antilouse metal comb. Then lice were picked up with forceps and placed in individual vials containing 95% Ethanol. A total of 75 human head lice samples were collected from 10 localities throughout the world (Table 1). Clothing lice came from two sites: Canada and Nepal. Canadian clothing lice (N=16) were collected from a single homeless person and provided to us by Douglas D. Colwell (Lethbridge Research Centre, Canada). The two clothing lice from Nepal (N=2) were collected from two persons and provided to us by Kazunori Yoshizawa (Hokkaido University, Japan). Vials were stored at -20° C.

***DNA extraction***

Lice were cut in half, placed into 1.5 ml Eppendorf tubes containing a cell lysis and proteinase K solution, and ground using a pestle. DNA was extracted from each individual louse using the Puregene Core Kit A (QIAGEN, Valencia, California) following manufacturer protocols with modifications. DNA concentration was determined using a NanoDrop 1000 spectrophotometer (Thermo Scientific). Samples were then diluted them to approximately 5-10 ng/μl.

### *Data harvesting, screen for repeat motifs and primer design*

Assembled genome sequence data for Pediculus humanus USDA strain (PhumU1, 2007) were obtained from VectorBase (<http://www.vectorbase.org/>). The clothing louse genome consists of 1,882 supercontigs totaling 110 Mb [1]. The genome was screened for tandem of perfect repeat motifs with a minimum of seven dinucleotide repeats, five tri-, and five tetranucleotide repeats, and a minimum suitable space of > 50 bp in the flanking 5’ and 3’ region for priming using msatcommander [2] or Tandem Repeat Finder [3]. Primer3Plus [4] was used to design primer pairs in flanking regions with a minimum 40% of GC content and 55°C melting temperature. Approximately, 150 primer pairs were designed and sequences were checked for duplication using BLAST (Basic Local Alignment Search Tool) [5,6]. We used the command-line BLAST version 2.2.26+ provided by the University of Florida Research Computing. To increase the sensitivity of BLAST searches against the human louse genome the default word size of eleven was changed to seven and the expect value cutoff parameter was set to 0.001.

***Single amplifications: Singleplex***

We used a cost-effective end-labeling of PCR products through a single-reaction nested or three primer PCR approach [7,8]. This method involves a fluorescently labeled universal primer in combination with modified locus-specific primers that have added 5′ universal primer sequence tails. The sequence tags correspond to M13 (5’-CACGACGTTGTAAAACGAC-3’) and CAG (5’-CAGTCGGGCGTCATCA-3’) [9,10]. M13-tag matches 6-FAM and VIC fluorescently labeled primers. NED and PET fluorescent primers were used with the CAG tagged primers. A PIG-tail sequence (5’-GCTTCT-3’) was incorporated to the 5’ end of some reverse primers to reduce stutters and facilitated scoring [11,12].

Three samples from different continents (America, Europe, and Asia) were used initially to test primer functionality. Microsatellites were amplified individually in 15-μl PCR reactions containing 7.5 μL of 2X Master Mix (Type-It Microsatellite PCR kit, Qiagen, Venlo, Netherlands), 0.015-0.15 μL of unlabelled forward primer with tail (10 μM), 0.15-0.8 of unlabelled reverse primer (10 μM), 0.5 μL labelled universal tail primer (10 μM), 1-2 μl of total genomic DNA (10-20 ng) and sdH_2_0 added up to final volume. Two thermal cycling profiles were used, both beginning with initial denaturation at 95°C (5 min) and ending with a final extension of 72°C (40 min). The v1 (touch-down PCR) protocol consisted of 10 cycles of 94°C (30 sec), 60°C → 55°C (-0.5°C/cycle) for 45 sec, and 72°C (45 sec) followed by 25 cycles of 94°C (30 sec), 55°C (45 sec), and 72°C (1 min). The v2 protocol consisted of 35 cycles of 94°C (30 sec), 52^o^C (45 sec), and 72°C (45 sec). PCR products were electrophoresed on 1.5% and 4% agarose gels stained with ethidium bromide, and visualized under ultraviolet light. Dilutions in sdH_2_0 from amplicons (1:100) were run on an ABI 3730xl 96-capillary sequencer using GeneScan 600 LIZ as an internal size standard (Applied Biosystems). Microsatellite genotypes were scored using the GeneMarker version 1.60 software (SoftGenetics, LLC). For scoring each locus, we used the function Create New Panel to outline the positions of alleles for each microsatellite marker in GeneMarker (SoftGenetics, LLC). Each panel included a set of bins that indicated specific base pair position for each allele. Panels were created using all the samples (Overlay Trace) and bins were manually edited. Roughly 33% (48) of the primer pairs produced amplicons of the expected size with a clear chromatogram and demonstrated allelic variation. These primer pairs were selected for further testing and multiplex optimization (Supplemental Table 1).

***Multiple co-amplification: Multiplexes***

To improve the high-throughput genotyping and limit the amount of louse DNA used in each PCR, we used a subset of primers to develop nine groups of 3-5 loci each for multiplex-ready PCR. Briefly, the multiplex combinations were selected based on: allele size ranges of the markers, universal primer sequence tails that allows for one or two fluorochromes, and relatively uniform amplification under the same PCR conditions when multiplexed.

First, primer pairs that produced amplicons of the expected size and demonstrated allelic variation were selected for further testing and multiplex optimization. Furthermore, we used one or two universal primers, each labeled with a unique fluorescent tag (e.g. FAM, VIC, NED, PET) to co-amplify multiple loci, including size-overlapping markers. First, primers were evaluated based on performance in each of the PCR protocols. This first evaluation allowed us to select group of primers that potentially could be group together in a multiplex-PCR. The second step was to use the help of the program MultiPLX 2.1 [13] in order to select each set of multiple primers. This program uses the nearest neighbor DNA binding thermodynamics to estimate possible unwanted pairings between PCR samples. The output contained groups of primers that were manually edited be removing some primers from each group to leave at least a total of 100 bp for each locus allele size range between loci. For example if a locus presented alleles: 120 to 130 bp, the potential total allele range for all worldwide populations would be considered: 80 to 180 bp. This criterion was thought in advance of our ongoing projects looking into the genetic diversity of human lice worldwide. A total of nine multiplexes were tested and included 27 pair of primers (Supplemental Table 1).

Optimizations of primer concentration and amplicon dilutions were conducted for the first four multiplexes (15 loci). To reduce pipetting errors, for each multiplex we prepared a single primer-master-mix where we combined all the primers (forward and reverse) that would be necessary for a little more than 4 plates (Supplemental Table 2). Final PCR-mixes for the four multiplexes can be found in Supplemental Table 3. Depending on amplicon intensity as observed from agarose gel, PCR products were diluted (1:75 to 1:200). Dilutions of amplicons were run on an ABI 3730xl 96-capillary sequencer using GeneScan 600 LIZ as an internal size standard (Applied Biosystems). Microsatellite genotypes were scored using the GeneMarker version 1.60 software (SoftGenetics, LLC). For the scoring of multiplexes, we created panels, one for each multiplex. In this case, each panel has three to four markers which each represent one locus. Within each marker a set of bins indicates the specific base pair position for each allele. The availability of these panels allows the software to automatically call the alleles. Proper allele calling was checked manually and adjusted where necessary. An overview of our primer development strategy and multiplex optimization is presented in Figure 2.

***Estimation of genotyping error***

Due to the small amount of DNA per louse, we use a repeat genotyping of sample method, to check genotyping quality. Five (5%) out the 93 samples were subject to a second round of multiplex-amplification. Afterwards allele sizes were compared and mistypes were counted. Mean error rate was estimated per locus, following Pompanon et al. [14] where e_l_ is the ratio between m_l_, the number of single-locus genotypes including at least one allelic mismatch, and n_t_, the number of replicated single-locus genotypes. In our study the average genotyping error rate per locus was 0.015. Error rates in the range 0.005-0.02 per locus have frequently been reported in the literature [14–16]. In addition no evidence for large-allele dropout for each locus in each population was detected using the software MICRO-CHECKER [17]. However, null alleles were suggested to be present in all the populations at many loci (see main manuscript).

***Mitochondrial PCR amplification and sequencing***

A polymerase chain reaction was performed to amplify the mitochondrial gene cytochrome c oxidase 1 (COX1) using the primers H7005 and L6625 [18] as described in [19]. PCRs consisted of 25 μL total volume including 10 μL of MasterMix (5 PRIME), 1 μL of each primer, 2-4 μL of total genomic DNA, and water. The thermal cycling profile began with an initial denaturation at 94°C (10 min) followed by 10 cycles of 94°C (1 min), 48°C (1 min), and 65°C (2 min) (decreased by 0.5°C per cycle). This was followed by 35 cycles of 94°C (1 min), 52°C (1 min), and 65°C (2 min) and then a final extension of 65°C (10 min). Amplified fragments were purified using ExoSAP-IT (USB Corporation, Cleveland, Ohio). Sequencing was performed at the University of Florida DNA Sequencing Core Laboratory (ICBR, Gainesville, Florida) using standard fluorescent cycle-sequencing PCR reactions (ABI Prism Big Dye terminator chemistry, Applied Biosystems).

***Sequence editing, alignment and haplotype reconstruction***

The forward and reverse sequences were edited and aligned using Sequencher 4.5 (Gene Codes Corporation, Ann Arbor, Michigan) with base calling confirmed by eye. Consensus sequences were generated for each sample using both forward and reverse sequences. The genetic relationships of all COX1 sequences were estimated by constructing neighbor-joining (NJ) trees [20] using PAUP* 4.0b10 [21]. Bootstrapping was performed using 100 pseudo-replications of the data set. This analysis provides unambiguous assignment of clade membership: A, B and C to each sequence [22].

**Supporting Information References**

1. Kirkness EF, Haas BJ, Sun W, Braig HR, Perotti MA, et al. (2010) Genome sequences of the human body louse and its primary endosymbiont provide insights into the permanent parasitic lifestyle. Proc Natl Acad Sci USA 107: 12168–12173. Available:http://www.ncbi.nlm.nih.gov/pubmed/20566863.

2. Faircloth BC (2008) msatcommander: detection of microsatellite repeat arrays and automated, locus-specific primer design. Mol Ecol Resour 8: 92–94. Available:http://www.ncbi.nlm.nih.gov/pubmed/21585724.

3. Benson G (1999) Tandem repeats finder: a program to analyze DNA sequences. Nucleic Acids Res 27: 573–580. Available:http://www.ncbi.nlm.nih.gov/pubmed/9862982.

4. Untergasser A, Nijveen H, Rao X, Bisseling T, Geurts R, et al. (2007) Primer3Plus, an enhanced web interface to Primer3. Nucleic Acids Res 35: W71–74. Available:http://www.ncbi.nlm.nih.gov/pubmed/17485472.

5. Altschul SF, Gish W, Miller W, Myers EW, Lipman DJ (1990) Basic local alignment search tool. J Mol Biol 215: 403–410. Available:http://www.ncbi.nlm.nih.gov/pubmed/2231712.

6. Altschul SF, Madden TL, Schäffer AA, Zhang J, Zhang Z, et al. (1997) Gapped BLAST and PSI-BLAST: a new generation of protein database search programs. Nucleic Acids Res 25: 3389–3402. Available:http://www.ncbi.nlm.nih.gov/pubmed/9254694.

7. Schuelke M (2000) An economic method for the fluorescent labeling of PCR fragments. Nat Biotechnol 18: 233–234. Available:http://www.ncbi.nlm.nih.gov/pubmed/10657137.

8. Blacket MJ, Robin C, Good RT, Lee SF, Miller AD (2012) Universal primers for fluorescent labelling of PCR fragments--an efficient and cost-effective approach to genotyping by fluorescence. Mol Ecol Resour 12: 456–463. Available:http://www.ncbi.nlm.nih.gov/pubmed/22268566.

9. Oetting WS, Lee HK, Flanders DJ, Wiesner GL, Sellers TA, et al. (1995) Linkage analysis with multiplexed short tandem repeat polymorphisms using infrared fluorescence and M13 tailed primers. Genomics 30: 450–458. Available:http://www.ncbi.nlm.nih.gov/pubmed/8825630.

10. Boutin-Ganache I, Raposo M, Raymond M, Deschepper CF (2001) M13-tailed primers improve the readability and usability of microsatellite analyses performed with two different allele-sizing methods. BioTechniques 31: 24–26, 28. Available:http://www.ncbi.nlm.nih.gov/pubmed/11464515.

11. Brownstein MJ, Carpten JD, Smith JR (1996) Modulation of non-templated nucleotide addition by Taq DNA polymerase: primer modifications that facilitate genotyping. BioTechniques 20: 1004–1006, 1008–1010. Available:http://www.ncbi.nlm.nih.gov/pubmed/8780871.

12. Ballard LW, Adams PS, Bao Y, Bartley D, Bintzler D, et al. (2002) Strategies for genotyping: Effectiveness of tailing primers to increase accuracy in short tandem repeat determinations. J Biomol Tech 13: 20–29. Available:http://www.ncbi.nlm.nih.gov/pubmed/19498960.

13. Kaplinski L, Remm M (2007) MultiPLX: automatic grouping and evaluation of PCR primers. Methods Mol Biol 402: 287–304. Available:http://www.ncbi.nlm.nih.gov/pubmed/17951801.

14. Pompanon F, Bonin A, Bellemain E, Taberlet P (2005) Genotyping errors: Causes, consequences and solutions. Nat Rev Genet 6: 847–859.

15. Bonin A, Bellemain E, Bronken Eidesen P, Pompanon F, Brochman C, et al. (2004) How to track and assess genotyping errors in population genetics studies. Mol Ecol 13: 3261–3273.

16. Hoffman JI, Amos W (2005) Microsatellite genotyping errors: detection approaches, common sources and consequences for paternal exclusion. Mol Ecol 14: 599–612. Available:http://dx.doi.org/10.1111/j.1365-294X.2004.02419.x.

17. Van Oosterhout C, Hutchinson WF, Wills DPM, Shipley P (2004) MICRO-CHECKER: software for identifying and correcting genotyping errors in microsatellite data. Mol Ecol 4: 535–538. Available:http://dx.doi.org/10.1111/j.1471-8286.2004.00684.x.

18. Hafner MS, Sudman PD, Villablanca FX, Spradling TA, Demastes JW, et al. (1994) Disparate rates of molecular evolution in cospeciating hosts and parasites. Science 265: 1087–1090. Available:http://www.ncbi.nlm.nih.gov/pubmed/8066445.

19. Reed DL, Smith VS, Hammond SL, Rogers AR, Clayton DH (2004) Genetic analysis of lice supports direct contact between modern and archaic humans. PLoS Biol 2: e340. doi:10.1371/journal.pbio.0020340.

20. Saitou N, Nei M (1987) The neighbor-joining method: a new method for reconstructing phylogenetic trees. Mol Biol Evol 4: 406–425.

21. Swofford DL (2003) Phylogenetic Analysis Using Parsimony (*and Other Methods). Sunderland, Massachusetts: Sinauer Associates.

22. Light JE, Allen JM, Long LM, Carter TE, Barrow L, et al. (2008) Geographic distributions and origins of human head lice (*Pediculus humanus capitis*) based on mitochondrial data. J Parasitol 94: 1275–1281. Available:http://www.bioone.org/doi/abs/10.1645/GE-1618.1.

23. Queller DC, Goodnight KF (1989) Estimating relatedness using genetic markers. Evolution 43: 258–275.
